# Ethylene Production Affects Blueberry Fruit Texture and Storability

**DOI:** 10.3389/fpls.2022.813863

**Published:** 2022-03-25

**Authors:** Brian Farneti, Iuliia Khomenko, Matteo Ajelli, Francesco Emanuelli, Franco Biasioli, Lara Giongo

**Affiliations:** ^1^Berries Genetics and Breeding Unit, Research and Innovation Centre, Fondazione Edmund Mach, Trento, Italy; ^2^Sensory Quality Unit, Research and Innovation Centre, Fondazione Edmund Mach, Trento, Italy

**Keywords:** *Vaccinium* spp., SRI-ToF-MS, texture analyzer, germplasm, biparental population

## Abstract

Ethylene, produced endogenously by plants and their organs, can induce a wide array of physiological responses even at very low concentrations. Nevertheless, the role of ethylene in regulating blueberry (*Vaccinium* spp.) ripening and storability is still unclear although an increase in ethylene production has been observed in several studies during blueberry ripening. To overcome this issue, we evaluated the endogenous ethylene production of a *Vaccinium* germplasm selection at different fruit ripening stages and after cold storage, considering also textural modifications. Ethylene and texture were further assessed also on a bi-parental full-sib population of 124 accessions obtained by the crossing between “Draper” and “Biloxi”, two cultivars characterized by a different chilling requirement and storability performances. Our results were compared with an extensive literature research, carried out to collect all accessible information on published works related to Vaccinium ethylene production and sensitivity. Results of this study illustrate a likely role of ethylene in regulating blueberry shelf life. However, a generalisation valid for all *Vaccinium* species is not attainable because of the high variability in ethylene production between genotypes, which is strictly genotype-specific. These differences in ethylene production are related with blueberry fruit storage performances based on textural alterations. Specifically, blueberry accessions characterized by the highest ethylene production had a more severe texture decay during storage. Our results support the possibility of tailoring *ad hoc* preharvest and postharvest strategies to extend blueberry shelf life and quality according with the endogenous ethylene production level of each cultivar.

## Introduction

Blueberry became, in the last decade, the second most important cultivated soft fruit species with a worldwide production that increased by almost 80% (Romo-Muñoz et al., 2019). The postharvest behavior knowledge specific for different blueberry accessions, including information on prolonged storage, is fundamental to guarantee year-round supply to cover the rising market demand, with shipped fruit that should ensure the best conditions upon delivery.

Despite a longer storability than other soft berries, blueberry’s susceptibility to decay, caused for the most part by *Botrytis cinerea* infection (Rivera et al., 2013), can enhance fruit perishability and greatly reduces shelf life (Cappellini et al., 1982). Blueberry shelf-life and organoleptic quality can be also negatively affected by fruit weight loss that enhances softening and skin wrinkling (Chiabrando and Giacalone, 2011; Paniagua et al., 2013; Perkins-Veazie, 2016). Indeed, a firm texture is one of the consumers’ most appreciated features, being associated with the general concept of blueberry freshness and quality (Ehlenfeldt and Martin, 2002; Gilbert et al., 2014; Daniels et al., 2018). In addition, blueberry fruit is susceptible to loss of integrity in the internal tissues (Allan-Wojtas et al., 2001), leading to discoloration as a dark blue or cell death as a red-brown. Therefore, appropriate postharvest techniques, properly tailored to extend storage of blueberry accessions, are needed.

Fruit storability and marketability are highly affected by an elevate ripening heterogeneity among fruit at harvest. During development, blueberry fruit mature at different rates, resulting in a non-uniform ripening period extending over two to 3 weeks (Suzuki et al., 1997b). Berries are considered ready to pick when they turn 100% blue, but since they do not ripen uniformly on a cluster (Lobos et al., 2014) growers usually wait several days for blue color to accumulate in the plant in order to optimize harvesting labor management and reduce production costs (Lobos et al., 2018). This heterogeneity in fruit ripening might negatively impact both fruit storability and organoleptic quality (Retamales and Hancock, 2012). A feasible solution to improve both harvest efficiency and fruit storability, can be the application of plant growth regulators. However, this application has to be tailored to the distinct ripening behavior of each accession.

Fruit ripening is for the most part regulated by multiple plant hormones such as ethylene, abscisic acid, auxins, and jasmonates, whose production rate and interactions are regulated by both genetic and environmental factors (McAtee et al., 2013). However, there are only few studies about the physiological and biochemical regulation of *Vaccinium* spp. fruit ripening in comparison with other climacteric and nonclimacteric model fruit species, like apple or strawberry. Thus, the active role of ethylene in regulating blueberry ripening and development is still doubtful. Although a rise in respiration and ethylene production has been observed in several studies during *Vaccinium* spp. fruit ripening (Windus et al., 1976; Shimura et al., 1986; Suzuki et al., 1997b; Goto et al., 2013), this was not conclusive in many others (Frenkel, 1972; Abdallah et al., 1989; Özgen et al., 2002). Proofs of the non-climacteric blueberry behavior are supported by the active role of abscisic acid (ABA) on fruit ripening, coloration and anthocyanins accumulation (Buran et al., 2012; Zifkin et al., 2012; Karppinen et al., 2013; Wang et al., 2018b). Similar inconsistencies regard the role of ethylene in regulating blueberry storability. Available results about increasing the fruit exposure to ethylene, for instance with ethephon or ethylene application (Poole et al., 1998; Costa et al., 2018; Wang et al., 2018b; Xu et al., 2020), or reducing the possible effect of ethylene applying ethylene scrubbers (Wang et al., 2018a; Álvarez-Hernández et al., 2019), 1-MCP (DeLong et al., 2003; Brackmann et al., 2010; MacLean et al., 2011; Blaker and Olmstead, 2014; Deng et al., 2014; Tao et al., 2017; Xu and Liu, 2017; Xu et al., 2020; Xue et al., 2020), or ozone (Song et al., 2003), indicated contrasting results and did not elucidate the role of ethylene on blueberry ripening.

The genotypes used in these published studies might considerably differ based on ethylene level production and/or ethylene sensitivity. Thus, the objectives of this work are (i) to elucidate the role of ethylene in blueberry fruit ripening with an extensive literature research, carried out to collect all accessible information on published works related to *Vaccinium* ethylene production and sensitivity; (ii) to verify the variability in ethylene production among a *Vaccinium* genotype collection during fruit ripening and storage; (iii) to verify a possible relation between the endogenous ethylene production and fruit textural variations during storage.

## Materials and Methods

### Literature Research About the Role of Ethylene in Regulating Quality and Storability of *Vaccinium* Species

An extensive literature search was carried out to collect all accessible information on published works related to *Vaccinium* ethylene production and sensitivity in Scopus and Web of Science databases. The name of the specific fruit (“Blueberr*” OR “Bilberr*” OR “Cranberr*”) and its botanical genus (“*Vaccinium*”) along with some keywords (“climacteric” OR “non-climacteric” OR “ethylene” OR “ethephon” OR “1-methylcyclopropene” OR “1-MCP”) were applied. Keywords were used in the fields [Topic] and [Article Title/Abstract/Keywords] for Web of Science and Scopus, respectively. All searches were carried out in April 2021. Only papers with, at least, the abstract in English were considered eligible with no restriction on the date of publication. A first selection of papers was performed according to abstract and title relevance. Full texts were obtained for the selected articles, and they were further assessed for appropriateness according to their relevance in determining a possible role of ethylene on *Vaccinium* fruit ripening and storability. If possible, recorded values of ethylene production rates were converted into ul kg^−1^ h^−1^.

### Plant Material and Fruit Sampling

Twenty-four *Vaccinium* accessions (Supplementary Table 1) were chosen from the collection experimental field of Fondazione Edmund Mach at Pergine (Trento), located in the North of Italy (Trentino Alto Adige region). At the time of the analysis, plants were in the full production phase, between 7 and 10 years old. Bushes were maintained following standard pruning and surface bark mulching renewal. In the plot, each of the accessions was represented at least by five plants.

Fruit were harvested at the commercial harvest maturity stage (Dark blue; DB). Homogeneous fruit of each cultivar, free from external damages or irregularities, were sampled immediately at harvest and divided into two batches of around 40 fruit each. Ethylene analyses were carried out at harvest, after 4 weeks of storage, at 2°C (RH 85%), and after one additional day of storage at 21°C.

Twelve of these 24 blueberry accessions (Supplementary Table 1) were chosen for an additional ethylene assessment during fruit ripening. Approximately twenty fruit for each cultivar., free from external damages or irregularities, were harvested at five ripening stages based on berry color: green (Gr), breaker (Br), red (Rd), blue (Bl), and dark-blue (DB). Ethylene analyses were carried after 3 h from harvest, with fruit preserved at 21°C.

Ethylene was further assessed on a bi-parental full-sib population of 124 accessions obtained by the crossing between “Draper” and “Biloxi”, two cultivars characterized, respectively, by a high and low -chill requirement. In this experimental design, seedlings from the progeny are represented by single plant of 3 years old. Bushes, grown on 35-L fertigated pots, were maintained following standard pruning and without disease control. Fruit were harvested at the commercial harvest maturity stage (Dark blue; DB) when the 75% of fruit, of the individual accession, were ripe. Homogeneous fruit of each accession, free from external damages or irregularities, were sampled immediately after harvest and divided into two batches of about 40 fruit each. Ethylene analyses were carried out for 4 h after harvest and after 4 weeks of storage at 2°C (RH 85%).

### Ethylene Analysis by PTR/SRI-ToF-MS

Ethylene measurements were performed with a commercial PTR-ToF-MS 8000 apparatus [Ionicon Analytik GmbH, Innsbruck (Austria)] equipped with switchable reagent ion system (SRI-ToF-MS) that allowed the operation of the instrument in H_3_O^+^ and O_2_^+^ modes, as described by Cappellin et al. (2014). The drift tube conditions were as follows: 110°C drift tube temperature, 2.25 mbar drift pressure, 550 V drift voltage. This leads to an E/N ratio of about 140 Townsend (Td), with E corresponding to the electric field strength and *N* to the gas number density (1 Td = 10^−17^ Vcm^2^). The sampling time per channel of ToF acquisition was 0.1 ns, amounting to 350,000 channels for a mass spectrum ranging up to *m/z* = 400. The sample headspace was withdrawn through SRI-ToF-MS inlet with 40 ml min^−1^ flow for 60 cycles resulting in an analysis time of 60 s/sample.

A custom built calibration system based on flow controllers (MKS, Andover, MA) was used to dilute ethylene standard (1 ppm_v_) with zero air (generated by a Gas Calibration Unit, Ionicon Analytik GmbH). Measurements were done with the SRI-ToF-MS in O_2_^+^ mode.

### Ethylene Assessment on Intact Berries

In order to measure the ethylene production of intact fruit during ripening and storage, three berries, after 4 h of equilibrium at 21°C, were put into a closed glass vial of 40 ml volume equipped with PTFE/silicone septa (Agilent, Cernusco sul Naviglio, Italy) and incubated at 21°C for 30 min. Each vial was then connected to the GCU zero air provider and to the SRI-ToF-MS operated in O_2_^+^mode. Concentration values of the masses *m/z* 26.012, *m/z* 28.031, *m/z* 29.034, *m/z* 42.011 were summed and converted into ethylene production rate (ul kg^−1^ h^−1^) based on the calibration curve (Supplementary Figure 1). Each measurement was done in four replicates.

### High Throughput Assessment of Ethylene on Powdered Frozen Material

Ethylene content was additionally estimated on powdered frozen material by setting the SRI-ToF-MS in H_3_O^+^mode (PTR-ToF-MS mode, Cappellin et al., 2014; Busatto et al., 2022). According with the recent publication about the application of a high-throughput methodology to assess blueberry VOC profile based on PTR-ToF-MS (Farneti et al., 2020), we analyzed the possibility to apply that methodology to measure ethylene, together with all other VOCs. Three replicates of 0.5 g of powdered frozen sample, each obtained by three fruit, were inserted into 20 ml glass vials equipped with PTFE/silicone septa (Agilent, Cernusco sul Naviglio, Italy) and mixed with 0.5 ml of deionized water, 200 mg of sodium chloride, 2.5 mg of ascorbic acid, and 2.5 mg of citric acid (Farneti et al., 2020). Sample headspace was withdrawn through SRI-ToF-MS (in H_3_O^+^mode) inlet with 40 ml min^−1^ flow for 60 cycles resulting in an analysis time of 60 s/sample. Pure nitrogen was flushed continuously through the vial to prevent any pressure drop. Each measurement was performed automatically after 20 min of sample incubation at 40°C and 2 min between each measurement was applied in order to prevent any memory effect. All measurement steps were automated by an adapted GC autosampler (MPS Multipurpose Sampler, GERSTEL) coupled to SRI-ToF-MS. Ethylene content was expressed as concentration (ul L^−1^) of the mass *m/z* 28.031.

This methodology, based on the analysis of powdered frozen material, was firstly applied on the same fruit that were previously measured non-destructively using the O_2_^+^ mode (blueberry cultivars assessed at different ripening stages and after storage; section Literature Research About the Role of Ethylene in Regulating Quality and Storability of *Vaccinium* Species). Secondly, it was applied on 124 accessions of the bi-parental population. Each accession was measured in triplicate.

### Texture Analysis

Texture was assessed immediately after storage on 10 homogenous fruit of each accession of both cultivars selection and bi-parental population by a texture analyzer (Zwick Roell, Ulm, Germany) equipped with a 5 kg loading cell and a cylindrical flat head probe with a diameter of 4 mm entering into the berry flesh from the sagittal side (Giongo et al., 2013, 2022; Farneti et al., 2020). The mechanical profile was defined by two variables: force (*N*) and distance (strain, %). The force was measured with the following instrumental settings: test speed of 100 mm min^−1^, post-test speed of 300 mm min^−1^, auto force trigger of 2 g, and stop plot at target position. Each berry was compressed until reaching the deformation of 90%. Based on the force displacement profile, three parameters were here considered: maximum force, deformation at maximum force and gradient (or Apparent Young’s module). All data were processed by TaxtExpertII software (Zwick Roell, Ulm, Germany).

### Data Analysis

The analysis of SRI-ToF–MS spectra proceeded as described in Cappellin et al. (2011, 2012). Peak intensities of each mass spectra were converted into concentrations (ul L^−1^) according to Lindinger et al. (1998). The data acquisition and extraction carried out on the texture mechanical profiles was operated by the software Exponent v.4 (Stable MicroSystem, Ltd., Godalming, United Kingdom) provided with the Zwick instrument. Data analysis was performed with R.4.0.2 software using internal functions and the external package “ggplot2” for graphic representations, and “splines” for polynomial regression model. For the polynomial regression model between ethylene and texture traits, knots were places at the lower, median quartile, and upper quartile of ethylene content/production. ANOVA levels of significance were set at *p* < 0.05 (*) and *p* < 0.01 (**).

## Results

### Bibliographic Research on *Vaccinium* Ethylene Production

The bibliographic research, carried out without any limitation about publication date, allowed to select 41 articles concerning the possible role of ethylene in regulating the ripening and senescence of V*accinium* fruit (Table 1). In total, 45 cultivars of different *Vaccinium* species were considered: *V. virgatum* (seven cv.), *V. corymbosum* (31 cv.), *V. corymbosum* x *V. darrowii* (three cv.) and *V. macrocarpon* (four cv.). For one article (Frenkel, 1972), neither the cultivar name nor the species were indicated. Within these studies, the number of trials carried out during pre-harvest and post-harvest phases were almost equivalent (35 and 39, respectively; as a “trial” we mean the combination of each treatment and each cultivars). More in detail, treatments with ethephon or ethylene were used to study the possible physiological and biochemical effects in 18 trials, while 19 trials addressed the effect of ethylene reduction by using 1-MCP post-harvest treatments, ozone application, or ethylene scrubbers (Table 1).

**Table 1:** Bibliographic research about published studies related to ethylene production and sensitivity of *Vaccinium* accessions.

*Vaccinium* spp	Cultivar	Ripening	Storage	Ethylene ul kg^−1^ h^−1^	Treatment to increase ethylene	Treatment to decrease ethylene	Notes	References
n.i	n.i.	X					Ethylene content is stable. blueberry fruit is not climacteric	Frenkel, 1972
*V. virgatum*	Austin		X	0,2			1-MCP application stimulated ethylene production, accelerated rate of firmness loss, and an increased TSS	MacLean et al., 2011
*V. virgatum*	Bluegem		X	2,5		X	1-MCP application in the storage room prolongs fruit storability	Brackmann et al., 2010
*V. virgatum*	Brightwell		X	0,4		X	1-MCP application stimulated ethylene production, accelerated rate of firmness loss, and an increased TSS	MacLean et al., 2011
*V. virgatum*	Brightwell		X	2,9		X	1-MCP application maintains fruit texture	Deng et al., 2014
*V. virgatum*	Powderblue	X			X		Ethephon application accelerates ripening in rabbiteye blueberry fruit, decreasing in the number of fruit harvests	Wang et al., 2018a
*V. virgatum*	Premier		X	0,7		X	1-MCP application stimulated ethylene production, accelerated rate of firmness loss, and an increased TSS	MacLean et al., 2011
*V. virgatum*	Premier	X			X		Ethephon application accelerates ripening in rabbiteye blueberry fruit, decreasing in the number of fruit harvests	Wang et al., 2018a
*V. virgatum*	Tifblue	X		3.7 mg/l			Respiratory climacteric at the reddish-green stage	Lipe, 1978
*V. virgatum*	Tifblue	X		3			Ethylene evolution peaked at the same time or a little earlier than respiration depending on the cultivar.	Shimura et al., 1986
*V. virgatum*	Woodard	X		6			Ethylene evolution peaked at the same time or a little earlier than respiration depending on the cultivar.	Shimura et al., 1986
*V. corymbosum*	Aurora						Differential expression of 39 genes related with ethylene	Gao et al., 2016
*V. corymbosum*	Berkeley	X		3			Ethylene increasing during fruit ripening	Suzuki et al., 1997b
*V. corymbosum*	Berkeley	X		3			Ethylene evolution peaked at the same or one color stage earlier than the ABA concentration	Goto et al., 2013
*V. corymbosum*	Berkeley		X	3		X	1-MCP plus UV-C irradiation treatment maintained postharvest quality and extended fruit storage	Xu and Liu, 2017
*V. corymbosum*	Berkeley		X	3,5	X	X	Ethylene treatment accelerates deterioration, and it improves ethylene production and respiration rate. 1-MCP application neutralize this accelerated ripening.	Xu et al., 2020
*V. corymbosum*	Berkeley		X	0,7 nmol/kg/s		X	Pressurized inert gas treatments with argon, carbon dioxide, and nitrogen suppressed the rate of respiration and ethylene production.	Meng et al., 2020
*V. corymbosum*	Berkeley		X	3,5			Mechanical vibration accelerated deterioration, which reflected in increasing ethylene production and respiratory rate	Xu et al., 2021
*V. corymbosum*	Bluecrop	X		0,4			Respiratory climacteric at the green-pink stage	Windus et al., 1976
*V. corymbosum*	Bluecrop		X		X		Postharvest treatment with ethylene enhanced anthocyanin content and antioxidant	Costa et al., 2014
*V. corymbosum*	Bluecrop		X	3			Ethylene content depends on storage time and on irradiation treatment	Wang et al., 2017
*V. corymbosum*	Bluecrop		X		X		The effect of ethylene treatment on the total anthocyanins and the total antioxidant activity is cultivar dependent	Costa et al., 2018
*V. corymbosum*	Blueray	X					Respiratory climacteric at the green-pink stage	Windus et al., 1976
*V. corymbosum*	Brigitta	X		1			Ethylene differences between cultivars. Peak of ethylene at 50% pink fruit	Moggia et al., 2018
*V. corymbosum*	Brigitta	X		0,2			Significant differences of ethylene content between cultivars, ripening stage and plant orientation	Lobos et al., 2018
*V. corymbosum*	Burlington		X			X	1-MCP application (400 nl/l) did not affect the fruit shelf life quality	DeLong et al., 2003
*V. corymbosum*	Collins	X		5			Ethylene increasing during fruit ripening	Suzuki et al., 1997b
*V. corymbosum*	Coville	X					Respiratory climacteric at the green-pink stage	Windus et al., 1976
*V. corymbosum*	Coville	X			X		Ethylene increased fruit respiration	Janes et al., 1978
*V. corymbosum*	Coville		X			X	No differences were found in ethylene production after ozone treatment	Song et al., 2003
*V. corymbosum*	Coville		X			X	1-MCP application (400 nl/l) did not affect the fruit shelf life quality	DeLong et al., 2003
*V. corymbosum*	Darrow	X					Respiratory climacteric at the green-pink stage	Windus et al., 1976
*V. corymbosum*	Dixi	X		1			Ethylene increasing during fruit ripening	Suzuki et al., 1997b
*V. corymbosum*	Duke	X		2,5			Ethylene differences between cultivars. Peak of ethylene at 50% pink fruit	Moggia et al., 2018
*V. corymbosum*	Duke	X		1,5			Significant differences of ethylene content between cultivars, ripening stage, and plant orientation	Lobos et al., 2018
*V. corymbosum*	Duke		X	4,2		X	Ethylene scrubber inhibits fungal decay incidence and extends the shelf life on fresh blueberry	Álvarez-Hernández et al., 2019
*V. corymbosum*	Duke		X		X		Ethylene has a crucial role in the acceleration of blueberry softening and sucrose degradation.	Wang et al., 2020
*V. corymbosum*	Earliblue	X					Respiratory climacteric at the green-pink stage	Windus et al., 1976
*V. corymbosum*	Earliblue	X			X		Ethylene increased fruit respiration	Janes et al., 1978
*V. corymbosum*	Earliblue	X		2,5			Ethylene evolution peaked at the same or one color stage earlier than the ABA concentration	Goto et al., 2013
*V. corymbosum*	Farthing		X	2			Ethylene differences between cultivars depend on storage conditions	Sargent et al., 2017
*V. corymbosum*	Goldtraube		X		X		Postharvest treatment with ethylene enhanced anthocyanin content and antioxidant	Costa et al., 2014
*V. corymbosum*	Goldtraube		X		X		The effect of ethylene treatment on the total anthocyanins and the total antioxidant activity is cultivar dependent	Costa et al., 2018
*V. corymbosum*	Herbert	X					Respiratory climacteric at the green-pink stage	Windus et al., 1976
*V. corymbosum*	Jersey		X		X		Ethylene treatment (10 ul ethylene/L) stimulated the anthocyanin synthesis	Paz et al., 1982
*V. corymbosum*	Jersey	X		3			Ethylene evolution peaked at the same time or a little earlier than respiration depending on the cultivar.	Shimura et al., 1986
*V. corymbosum*	Jersey	X		2–7,5			ACC treatment of immature berry increased ethylene evolution and accelerated maturation.	Suzuki et al., 1997a
*V. corymbosum*	Jersey	X		1,5			Ethylene evolution peaked at the same or one color stage earlier than the ABA concentration	Goto et al., 2013
*V. corymbosum*	Jersey	X		2,5			Ethylene, not ABA, induced maturation of blueberry at the mature green stage	Watanabe et al., 2021
*V. corymbosum*	Lanfeng		X	3,5		X	Ethylene Absorbent inhibit fruit softening and quality deterioration, and reduce the loss of total phenolic contents.	Wang et al., 2018b
*V. corymbosum*	Lateblue	X		0,5			Respiratory climacteric at the green-pink stage	Windus et al., 1976
*V. corymbosum*	Legacy		X			X	MAP+1-MCP treatment delayed fruit softening, inhibit the increase of fruit respiration rate and ethylene production rate.	Xue et al., 2020
*V. corymbosum*	Lenoir	X					High expression of ACC oxidase gene CUFF.81159, suggested that ethylene is produced throughout berry fruit development	Gupta et al., 2014
*V. corymbosum*	North Land		X			X	1-MCP improved the storability of blueberry fruit	Tao et al., 2017
*V. corymbosum*	Nui		X		X		Exogenous ethylene application stimulated ACC and ethylene content	Poole et al., 1998
*V. corymbosum*	O”Neal	X					High expression of ACC oxidase gene CUFF.81159, suggested that ethylene is produced throughout berry fruit development	Gupta et al., 2014
*V. corymbosum*	O”Neal		X			X	1-MCP improved the storability of blueberry fruit	Tao et al., 2017
*V. corymbosum*	Ozark Blue	X					High expression of ACC oxidase gene CUFF.81159, suggested that ethylene is produced throughout berry fruit development	Gupta et al., 2014
*V. corymbosum*	Ozark Blue		X		X		Postharvest treatment with ethylene enhanced anthocyanin content and antioxidant	Costa et al., 2014
*V. corymbosum*	Ozark Blue		X		X		The effect of ethylene treatment on the total anthocyanins and the total antioxidant activity is cultivar dependent	Costa et al., 2018
*V. corymbosum*	Pamlico	X					High expression of ACC oxidase gene CUFF.81159, suggested that ethylene is produced throughout berry fruit development	Gupta et al., 2014
*V. corymbosum*	Puru		X		X		Exogenous ethylene application stimulated ACC and ethylene content	Poole et al., 1998
*V. corymbosum*	Reka		X		X		Exogenous ethylene application stimulated ACC and ethylene content	Poole et al., 1998
*V. corymbosum*	Star		X			X	1-MCP application 5 days prior to harvest decreased fruit firmness of southern highbush blueberries at the time of harvest	Blaker and Olmstead, 2014
*V. corymbosum*	Sweetcrisp		X			X	1-MCP application 5 days prior to harvest decreased fruit firmness of southern highbush blueberries at the time of harvest	Blaker and Olmstead, 2014
*V. corymbosum*	Windsor		X	0,8			Ethylene differences between cultivars depend on storage conditions	Sargent et al., 2017
*V. corymbosum x V. darrowii*	Emerald		X			X	Pre-treatment with 1-MCP alleviated MeBr induced internal breakdown, firmness loss and wall degradation in blueberry.	Ortiz et al., 2018
*V. corymbosum x V. darrowii*	Jewel		X			X	Pre-treatment with 1-MCP alleviated MeBr induced internal breakdown, firmness loss and wall degradation in blueberry.	Ortiz et al., 2018
*V. corymbosum*	Weymouth	X		2			Ethylene evolution peaked at the same time or a little earlier than respiration depending on the cultivar.	Shimura et al., 1986
*V. darrowii x V. corymbosum*	JU83		X		X		Exogenous ethylene application stimulated ACC and ethylene content	Poole et al., 1998
*V. macrocarpon*	Early Black		X	7,8	X		Ethylene treatment promotes red color	Craker, 1971
*V. macrocarpon*	n.i.	X		3 ppm			Ethylene increased during fruit ripening	Forsyth and Hall, 1967
*V. macrocarpon*	Searles	X		0,3			The increase of ethylene does not coincide with CO_2_ production. Blueberry is not climacteric	Abdallah et al., 1989
*V. macrocarpon*	Stevens		X	1,7			Inhibition of ethylene production in cranberry fruits by lysophosphatidylethanolamine	Ryu et al., 1997
*V. macrocarpon*	Stevens		X	1 mg/kg/h			Rate of ethylene production did not vary significantly among ripeness stages	Özgen et al., 2002

*All accessions employed in these studied were sorted based on the species. Additional columns indicate if the study regarded fruit ripening or storage and if any treatment to increase or decrease the ethylene content were applied*.

In all studies, the most employed cultivars were “Berkeley” (six trials), “Jersey” (five trials), “Bluecrop” (four trials), “Coville” (four trials), “Duke” (four trials), “Ozark Blue” and “Earliblue” (three trials). Overall, trials carried out using the same cultivars (i.e., “Berkeley,” “Duke”, and “Jersey”) showed consistent results regarding both ethylene concentrations and physiological effect on ripening and storability, for instance on color and texture changes. Unfortunately, the exact ethylene production level was reported only on a limited number of articles (25 articles; 38 trials). The comparison between the ethylene production rates, reported in these papers, was not always possible since measurement units were not comparable (Forsyth and Hall, 1967; Lipe, 1978; Özgen et al., 2002; Meng et al., 2020). The high variability in both ethylene production levels and physiological responses of berries to ethylene treatment could be related with the high heterogeneity of species and cultivars employed in all these studies.

These studies indicated that, independently of the species, the *Vaccinium* accessions characterized by a significant increase in ethylene production during maturation and storage are also more sensitive to treatments applied to reduce and /or increase fruit exposure to ethylene. In particular, texture, color and nutraceutical content are the most influenced fruit quality traits.

### Variability in Ethylene Production During Fruit Ripening

Ethylene emission rate of 12 blueberry cultivars (“Atlantic,” “Berkeley,” “Biloxi,” “Bluechip,”“Bluegold,” Brigitta Blue,” “Chandler,” “Emerald,” “Jersey,” “Jubilee,” “Legacy,” and “Misty”; Supplementary Table 1) was evaluated at different fruit ripening stages (GR, green; BR, breaker; RD, red; BL, blue; DB, dark blue). The ethylene produced by each berry was measured using the SRI-ToF-MS applied in O_2_^+^ mode. The concentration of masses related with ethylene molecule (*m/z* 26.012, *m/z* 28.031, *m/z* 29.034, *m/z* 42.011) were quantified through a calibration curve obtained with a gas reference standard (Supplementary Figure 1).

**Figure 1:**
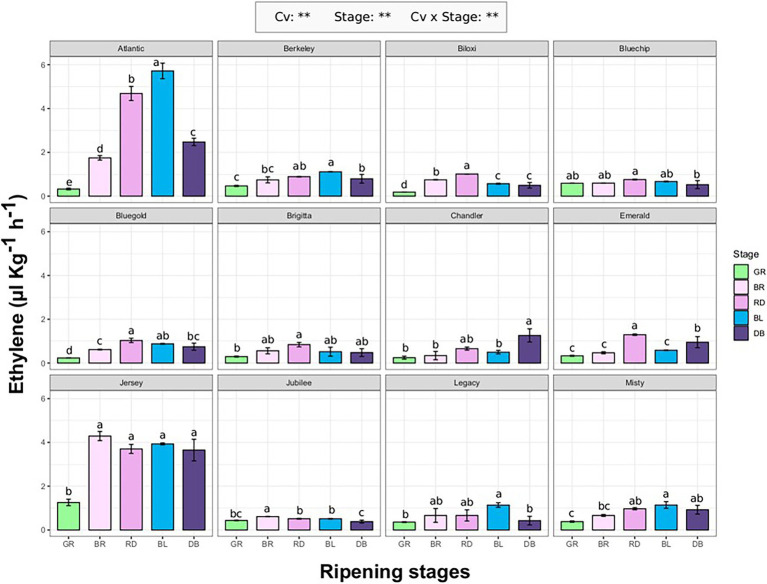
Ethylene production rate of 12 blueberry cultivars assessed at five fruit ripening stages: green (Gr), breaker (Br), red (Rd), blue (Bl), and dark-blue (DB). Each measurement was done in four biological replicates. Ethylene measurement was assessed on intact berries by using an PTR/SRI-ToF-MS set in O_2_^+^mode. ANOVA levels of significance (*p* < 0.05 and *p* < 0.01) are represented by ^*^ and ^**^, respectively. Different letters indicate significant differences between treatments (*p* ≤ 0.05) according to Tukeyʼs post-hoc test.

Ethylene production significantly differs (*p* < 0.01) among cultivars and fruit ripening stages (Figure 1). For most cultivars, maximum ethylene production corresponds with the berry veraison matching with RD and/or BL ripening stages. Next to the accomplishment of ripening (stage DB), the ethylene production tended to decrease and it returned to the values measured at the beginning of fruit veraison (stage BR). The only exception concerned “Jersey” which showed a high production of ethylene (about 4.2 ul kg^−1^ h^−1^) already at breaker stage (BR) which remained statistically unchanged until the complete ripening of the berry. “Jersey” and “Atlantic,” being the first parent of the second, were the two cultivars with the maximum ethylene rates recorded during ripening, respectively of 4.2 ul kg^−1^ h^−1^ and 5.8 ul kg^−1^ h^−1^. For the remaining cultivars, the maximum values were around 1 ul kg^−1^ h^−1^. There was no significant effect of type of blueberry cultivars (Northern high bush, NHB; Southern high bush, SHB; High bush, HB) on the production trend of ethylene during berry ripening (Supplementary Figure 2).

### Variability in Ethylene Production During Blueberry Storage

#### Ethylene Production of Commercial Blueberry Cultivars

**Figure 2:**
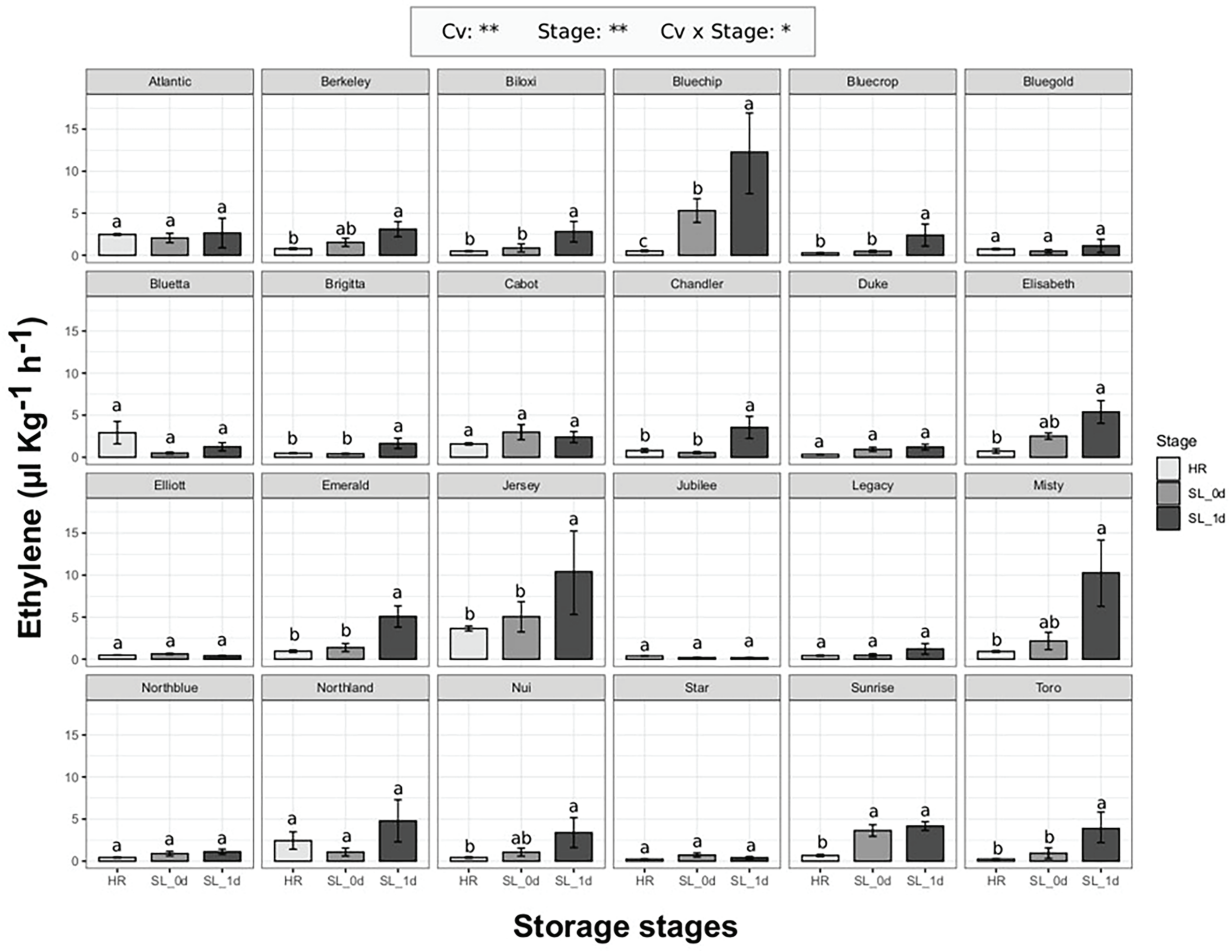
Ethylene production rate of 24 blueberry cultivars assessed at harvest (HR), after 4 weeks of storage at 2°C (SL_0d), and after one additional day of shelf life at 21°C (SL_1d). Each measurement was done in four replicates. Ethylene measurement was assessed on intact berries by using an PTR/SRI-ToF-MS set in O_2_^+^mode. ANOVA levels of significance (*p* < 0.05 and *p* < 0.01) are represented by ^*^ and ^**^, respectively. Different letters indicate significant differences between treatments (*p* ≤ 0.05) according to Tukeyʼs post-hoc test

The ethylene production of 24 blueberry cultivars (Supplementary Table 1) was evaluated at harvest (HR) when berries reached commercial maturity, and after 4 weeks of storage at 2°C, both immediately after storage (SL_0d) and after an additional 1 day of shelf life at 21°C (SL_1d). As previously reported on berries assessed at different ripening stages, ethylene production significantly (*p* < 0.01) differed among both cultivars and berry storage stages (Figure 2). It is possible to identify two cultivar groups: cultivars with an increased ethylene production during storage (13 cv. out of 24) and cultivars without any significant effect of storage on ethylene production. Among cultivars with a significant ethylene boost during storage, some accessions showed a remarkable increase of ethylene only after 1 day of shelf life such as “Bluechip” (about 12 ul kg^−1^ h^−1^), “Jersey” (about 10 ul kg^−1^ h^−1^), “Misty” (about 10 ul kg^−1^ h^−1^), “Emerald” (about 5 ul kg^−1^ h^−1^), “Toro” (about 4ul kg^−1^ h^−1^), “Chandler” (about 4ul kg^−1^ h^−1^). There was no significant effect of the earliness/lateness of the cultivars (harvest times, Supplementary Figure 3) on the detected ethylene production levels.

The production of ethylene during storage did not depend on ethylene release at harvest. Some cultivars, as “Atlantic,” “Bluetta” or “Cabot,” produced moderate values of ethylene at harvest that remained stable during storage. Other cultivars, like “Jersey,” further increased the ethylene production during storage despite the high levels detected at harvest. On the other hand, there were cultivars, such as “Bluegold,” “Elliott,” “Jubilee” or “Star,” characterized by a stable low ethylene production both at harvest and storage, and cultivars, as “Bluechip,” “Misty,” “Sunrise” or “Toro,” showing a rising ethylene content during storage despite the low levels assessed at harvest.

Results of this trial were further applied to test the use of SRI-ToF-MS (at H_3_O^+^ mode) combined with a multipurpose auto-sampler to evaluate the ethylene content on an aliquot of frozen and powdered fruit instead of the whole fruit. This high throughput methodology, previously developed for the analysis of blueberry volatilome (Farneti et al., 2020), aimed on a more qualitative characterization of the ethylene production, rather than a precise and absolute quantification. The ethylene content of the 24 blueberry cultivars, assessed on the headspace of intact berries at harvest and after storage, was correlated with the concentration of mass *m/z* 28.031 measured, destructively, on the same powdered frozen fruit (Supplementary Figure 4). The high correlation between the ethylene values measured with both methodologies suggested the possibility of using the concentration of the mass peak 28.031 (measured with the SRI-ToF-MS in H_3_O^+^ mode) to make a quick and qualitative estimation of ethylene content, even in combination with the entire fruit volatilome (data not shown). With this method, up to 350 samples a day can be automatically measured assessing both ethylene release and VOC fingerprint.

#### Ethylene Variability Between Accessions of a Biparental Population

The ethylene production of a bi-parental full-sib population obtained from the crossing of “Draper” x “Biloxi” (124 accessions), was evaluated on ripe berries at harvest and after 4 weeks of storage at 2°C, by using the high throughput methodology based on SRI-ToF-MS (at H_3_O^+^ mode), that we have previously described. For an additional validation of this high throughput methodology, the ethylene produced by the parental cultivars “Draper” and “Biloxi,” at different stages of ripening and storage, was measured applying both methodologies (Supplementary Figure 5).

**Figure 3:**
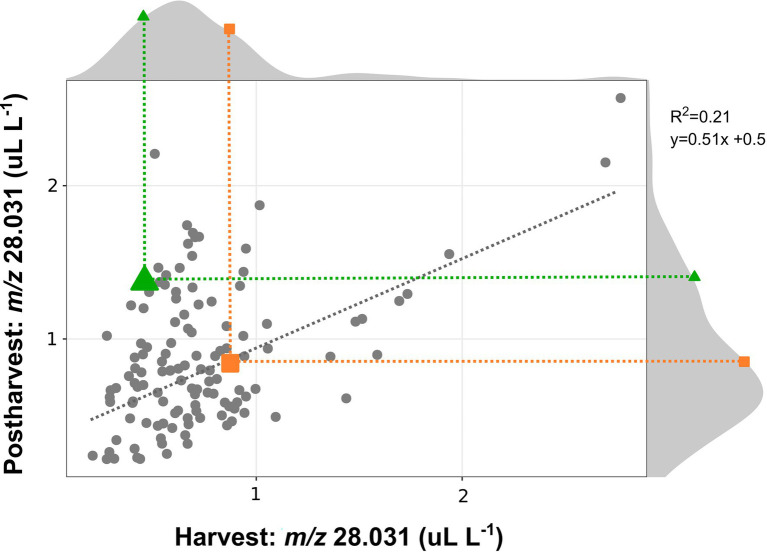
Correlation plot and density plot of the ethylene content determined at harvest and after 4 weeks of storage of 124 accessions of a bi-parental full-sib population obtained by the crossing between the cultivars “Biloxi” (green triangle) and “Draper” (orange square). Each measurement was done in three replicates. Ethylene measurement was assessed on frozen powdered material by using an PTR/SRI-ToF-MS in H_3_O^+^mode. Ethylene content was estimated taking into account the concentration of the mass *m/z* 28.031.

Data obtained on this segregating population (Figure 3) were coherent with the data collected on the 24 blueberry cultivars. These results confirmed a high genotype variability in the ethylene production of fruit analyzed both at harvest and after storage. In agreement with the above-mentioned results obtained on blueberry cultivars, the correlation between the ethylene content at harvest and after storage was quite low (*r*^2^ = 0.21) due to two distinct behavior patterns among all accessions: (i) accessions with similar ethylene content both at harvest and after storage, like “Draper”; (ii) accessions with an enhanced content of ethylene after storage, like “Biloxi.” especially for accessions characterized by a limited ethylene production at harvest. This correlation improved considering also the accessions characterized by a high production of ethylene at harvest (greater than 1 ul L^−1^). The ethylene production of the two parental cultivars, “Draper” and “Biloxi,” was similar at harvest. However, they showed two distinct behaviors after storage: the ethylene production was stable in “Draper” while it increased in “Biloxi.” This trend is also confirmed by the non-destructive analysis of the fruit headspace on these two parental cultivars (Supplementary Figure 5).

### Role of Ethylene in Regulating Berry Softening

**Figure 4:**
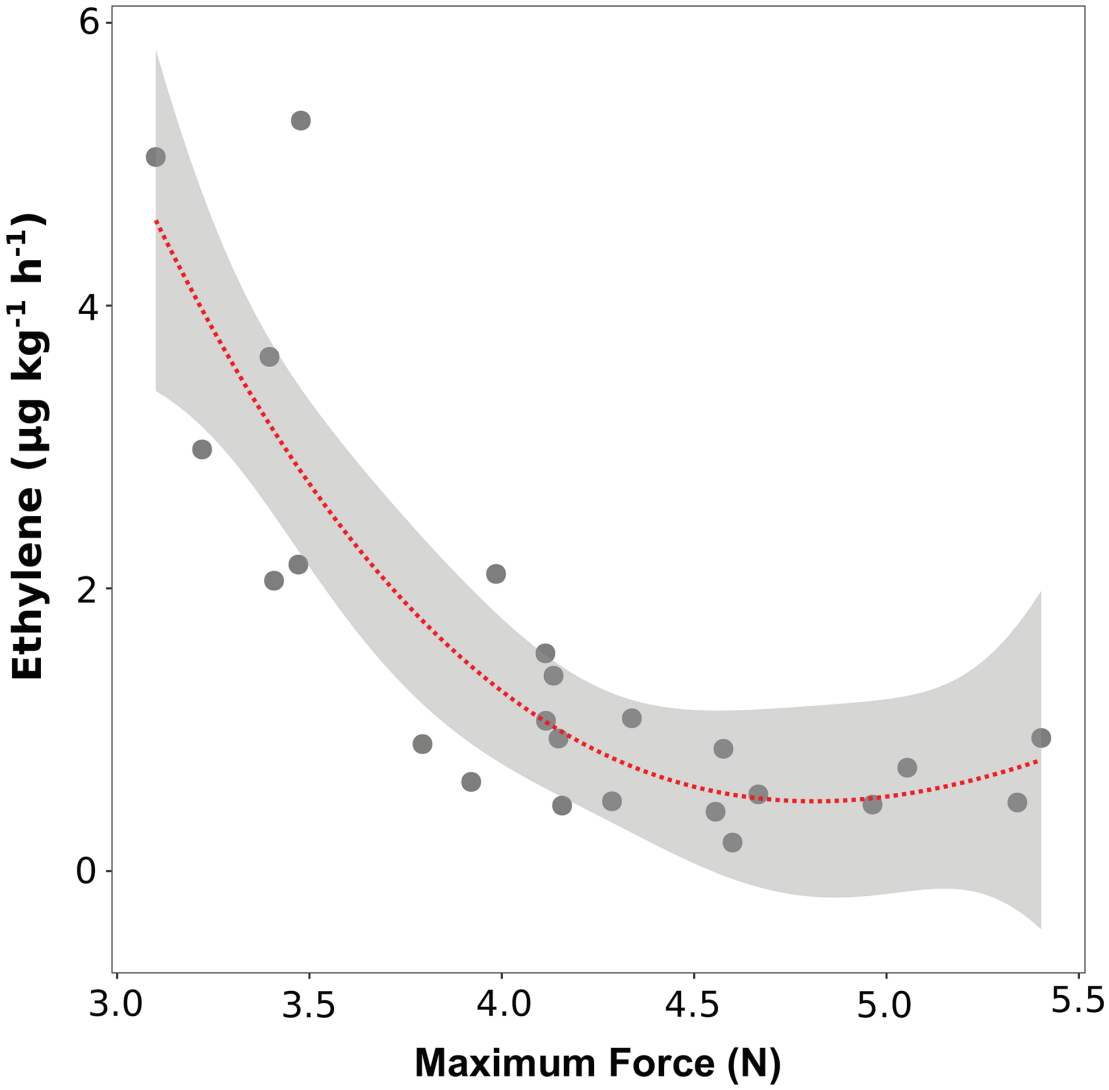
Correlation plot and polynomial regression model between the ethylene production and the textural value of maximal force of 24 blueberry cultivars assessed after 4 weeks of storage. Ethylene measurement was assessed in four replicates on intact berries by using an PTR/SRI-ToF-MS set in O_2_^+^mode. Texture analysis was assessed on 10 berries by using a texture analyzer.

To verify the role of ethylene in regulating fruit softening, we evaluated the link between ethylene content and fruit texture, before and after storage. At the time of harvest, no significant correlation was found between texture parameters (maximum force, Young’s modulus and deformation at maximum force) and ethylene content (data not shown). Immediately after storage (SL_0d), no significant linear correlations were highlighted for most accessions; however, six varieties (“Bluechip,” “Jersey,” “Sunrise,” “Cabot,” “Misty,” and “Atlantic”) defined by high ethylene productions (above 2 ul kg^−1^ h^−1^) were also characterized by the lowest maximum force values (lower than 3.5 N, Figure 4). No significant correlation was found between ethylene production and Young’s modulus and deformation at maximum force (Supplementary Figure 6).

**Figure 5:**
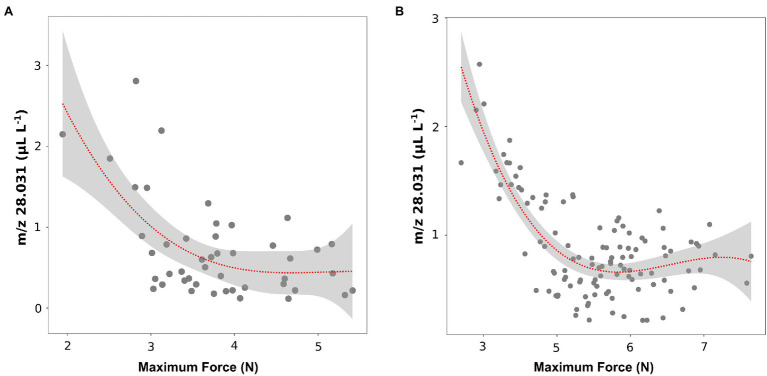
Correlation plot and polynomial regression model between the ethylene content and the textural value of maximal force of 46 blueberry cultivars **(A)** and 124 accessions of a bi-parental full-sib population **(B)** both assessed after storage. Ethylene measurement was assessed on frozen powdered material by using an PTR/SRI-ToF-MS set in H_3_O^+^mode. Texture analysis was assessed on 10 berries by using a texture analyzer. Results of plot A were extrapolated from the raw data of Farneti et al. (2020).

These results about texture parameters (maximum force) and ethylene concentrations (*m/z* 28.031) were coherent with results of the recent article by Farneti et al. (2020), in which the volatilome and texture of 46 *Vaccinium* cultivars were analyzed both at harvest and after 6 weeks of storage. Results obtained on berries assessed after storage confirm a possible relation between texture (maximum force values) and ethylene content (Figure 5A). However, after storage only six out of 46 cultivars (“Jersey,” “Jewell,” “Emerald,” “Safir,” “Azur,” “Atlantic”) showed, very low values of texture (maximum strength less than 3 N) and, at the same time, high values of ethylene (*m/z* 28.031 greater than 1.5 ul L^−1^).

These outcomes were further confirmed by the analysis of ethylene (*m/z* 28.031) and texture (maximum force) of the 124 accessions of the bi-parental full-sib segregating population (Figure 5B). Similar to the above-mentioned results, no significant linear correlation between ethylene and texture was observed considering the entire pool of genotypes. However, we identified a group of accessions characterized by a lower texture performance, with maximum strength values below 3.5 N, and, at the same time, a higher production of ethylene (greater than 1 ul L^−1^ of *m/z* 28.031).

## Discussion

Endogenous ethylene production by plants, can induce relevant physiological responses at concentrations as low as 0.1 μl L^−1^ (Watada, 1986). In particular, mechanisms of fruit ripening and senescence could involve both ethylene-dependent and ethylene-independent processes, which may differ in extent and/or manner depending on the species (Paul et al., 2012). Monitoring and manipulating fruit ethylene production, along with respiration levels, is therefore of paramount importance in establishing the optimal storage strategy.

Among soft berry species (i.e., strawberry, raspberry or blackberry), for blueberry there are some more scientific evidences on a possible active role of ethylene in both phases of ripening and storage (Table 1). However, the role of ethylene has not been yet fully clarified based on the very contrasting results reported in literature (Table 1), still keeping the dispute open whether the blueberry is climacteric or not. One of the causes of this controversy could lie in the use of different cultivars and species of *Vaccinium* (*V. virgatum*, *V. corymbosum*, *V. corymbosum x V. darrowii*, and *V. macrocarpon*; Table 1) in all the studies regarding fruit maturation and storage. Studies, in which the same cultivars were taken into consideration in different years and/or cultivation locations, show indeed comparable results (Table 1).

Results obtained in our study confirmed the high variability in ethylene production and release that can be found within the blueberry germplasm, and how this variability seems more related to genotype rather than species dependent. For instance, no differences in ethylene production were associated with the distinction between Northern highbush and Southern highbush (Figures 1, 2), as it has also been confirmed by the bibliographic research (Table 1). Results obtained by the analysis of a bi-parental full-sib population generated by the cross between “Draper” and “Biloxi” (Figure 3) suggested a direct genetic control of fruit ethylene production both at harvest and after storage.

Genotype related differences in ethylene production were evident during both fruit ripening and storage (Figures 1–3). Accessions characterized by a non-basal production of ethylene (such as “Atlantic,” “Jersey,” “Bluegold,” or “Biloxi”), showed an increase of ethylene production during fruit veraison, which tends to decrease with the progress of maturation, as previously confirmed by several studies (Windus et al., 1976; Shimura et al., 1986; Suzuki et al., 1997b; Goto et al., 2013). However, despite a regular ripening development of the fruit, this effect cannot be found on the accessions characterized by a constant trace production of ethylene (such as “Jubilee,” “Legacy,” “Bluechip,” or “Chandler”). This result suggests a subordinate role of ethylene in regulating blueberry fruit ripening. Another hypothesis, already suggested for non-climacteric fruits, such as grape (Chervin et al., 2004), supports the requirement of traces amounts of ethylene to start the ripening process. Such ethylene concentrations might be below the sensitivity threshold of the analytical technique used so far to quantify ethylene.

In several studies the colour and metabolic changes characterizing blueberry ripening are associated with a physiological role of abscisic acid (Karppinen et al., 2018; Oh et al., 2018) as for many other fruit species classified as non-climacteric (Bai et al., 2021). However, this hypothesis does not exclude a possible interaction between ethylene and abscisic acid, as it has already been found on strawberry fruit (Li et al., 2020; Tosetti et al., 2020). The colour changes of blueberries coincide with several biochemical changes related with the anthocyanin synthesis (Günther et al., 2020), the sugar/acid ratio change (Forney et al., 2012) and also with the variations in VOC profile (Farneti et al., 2017a). Such biochemical changes are associated with the up-regulation of genes involved in enzymatic activity, such as UDP-glucosyltransferases, an alcohol dehydrogenase, and hormone biosynthetic genes (Gupta et al., 2015). These metabolic changes related with fruit ripening are mostly independent from the production of ethylene, since they occur indistinctly from the level of ethylene produced by the fruit. However, this does not exclude a possible secondary role of ethylene in catalyzing these physiological processes. The often-conflicting results obtained after applying ethephon or 1-MCP during blueberry ripening, underline the need to develop chemical/agronomic strategies for a more precise control of fruit ripening, specific for each accession of blueberry.

Results of our study (Figures 2, 3) demonstrated that many blueberry accessions have a second post-harvest ethylene production increase that is probably associated with the senescence of the fruit or with an effect of exposure to cold temperature. For several accessions, the ethylene concentration, both at the time of release from the cold room and after 24 h of shelf life at room temperature, has fairly high values comparable to other fruit species commonly considered as climacteric, such as banana (around 1.5–2 ul kg^−1^ h^−1^; de Martino et al., 2007), tomato (around 5–8 ul kg^−1^ h^−1^; Yokotani et al., 2009) and kiwifruit (around 8–10 ul kg^−1^ h^−1^; Antunes and Sfakiotakis, 2002; Minas et al., 2018).

The magnitude of this second post-harvest ethylene increase seems to be inversely related to the blueberry texture, especially to the maximum force value (Figures 4, 5). Based on results obtained both on the varietal collection and on the segregating population (“Draper” x “Biloxi”), this correlation is evident only for the accessions characterised by a high ethylene production rate (higher than 2 ul kg^−1^ h^−1^). On the contrary, ethylene production was not significantly correlated with the values of Young’s modulus and deformability (Supplementary Figure 6). While blueberry fruit elasticity is a consequence of fruit turgor and cell wall strength (Fava et al., 2006; Rivera et al., 2021), fruit softening is a result of hemicellulosic depolymerization and pectic polysaccharides change (Vicente et al., 2007). Mechanical changes associated with berry softening observed during blueberry ripening and storage differ among species and cultivars and they are likely determined by modification in the content of different cell wall polymers (Vicente et al., 2007; Angeletti et al., 2010; Chen et al., 2015; Liu et al., 2019).

Ethylene regulates fruit softening of several climacteric plant species (Ireland et al., 2014). However, the role of ethylene in blueberry softening is still not clear as it has been confirmed by contrasting results of several studies (Table 1). Few articles (DeLong et al., 2003; Blaker and Olmstead 2014; Costa et al., 2014) do not show any significant effect of ethylene on fruit texture during ripening and storage, while others (Ortiz et al., 2018; Wang et al., 2020) suggest an active role of ethylene in regulating fruit softening rate. Postharvest technologies aimed at reducing environmental ethylene (i.e., KmNO4 scrubber) and its perception (i.e., 1-MCP) resulted in textural improvements (Chiabrando and Giacalone, 2011; Ortiz et al., 2018; Wang et al., 2018a; Xue et al., 2020; Xu et al., 2020). In particular, 1-MCP treatment delayed the hemicelluloses solubilization in blueberry fruit (cv. “Brightwell”) over the whole storage, and it decreased the Endo-beta-glucanase (EGase) activity (Deng et al., 2014). On the opposite, exogenous ethylene treatments can significantly promote the softening of blueberry fruit (cv. “Duke”) by promoting cell wall degradation *via* stimulation of PE, PG, and β-gal enzyme activities and VcPE and VcPG expression (Wang et al., 2020). However, blueberry fruit softening might be also regulated by other hormones, especially ABA. Indeed, post-harvest application of ABA enhanced the expression of many genes involved in cell wall disassembly in bilberry (*Vaccinium myrtillus*) fruit (Karppinen et al., 2018).

In addition to the possible regulation of blueberry texture, ethylene could also have an indirect role in controlling the fruit shelf life by inducing the development of fungal pathogens (i.e., *B. cinerea*). The active role of ethylene on regulating the growth of certain fungal and bacterial pathogens is well known (Cristescu et al., 2002; Zhu et al., 2012). Fungal (i.e., *B. cinerea*) germination and hyphal growth might be also promoted in blueberries due to ethylene presence (Chiabrando and Giacalone, 2011). Fungal (*B. cinerea*) decay incidence, on fresh blueberry stored under modified atmosphere packaging (MAP) conditions was significantly inhibited by using ethylene scrubbers consisting of a protonated montmorillonite loaded with KMnO_4_ (Álvarez-Hernández et al., 2019). However, it is not possible from these studies to establish whether any enhanced mold growth induced by ethylene is a secondary effect arising from increased cell permeability that facilitates germination and growth of fungal spores, or any inhibition of mold growth is due to a defence response by tissues to ethylene. Since botrytis is one of the main post-harvest pathogens of blueberry, results of our study recommend a greater attention in storage especially for blueberry accessions characterized by a more abundant production of ethylene. The application of ethylene scrubbers, especially in MAP, or treatments to reduce the concentration of ethylene (i.e., ozone or 1-MCP application) could have the synergic positive effect of slowing the decay of the berry texture and reducing the possible attack of fungal infections and will be further investigated.

This study illustrates a likely role of ethylene in regulating blueberry shelf life. However, a solid generalisation valid for all *Vaccinium* species is not attainable because of the high variability in ethylene production between genotypes, which is strictly genotype-specific. Up to these results, this high variability is the main cause of the discordant literature data on the role of ethylene in regulating blueberry ripening and storability. This does not exclude a possible role of other hormones, especially ABA, in fruit ripening and senescence. In addition, texture data, both on varietal collection and segregated population, highlight a possible active role of ethylene which, however, does not fully explain the high variability in texture between genotypes. The extreme variability in ethylene production between genotypes is common for many species considered strictly climacteric, such as apple (Farneti et al., 2017b), tomato (Zhao et al., 2021), melon (Zheng and Wolff, 2000; Pereira et al., 2020), or peach (Guo et al., 2018). This must be a warning about the excessive approximation in classifying the various fruit and vegetable species in climacteric and non-climacteric based exclusively on the ethylene and respiration average values extrapolated from few reference cultivars. To optimize pre- and post-harvest cultivation practices, suitable for a detailed and precise control of fruit ripening, it is recommended to obtain all possible information on ethylene production values of each accession of commercial interest. This can help to develop specific cultivation and storage strategies for each genotype in order to maximize the sustainability of the entire production chain focusing on a lower energy input, higher organoleptic quality of the product and a reduced product loss during storage. To this end, future breeding programs focused on prolonged fruit post-harvest storage need to also consider ethylene production. This can be achieved only with a more informed and precise identification of the best performing cultivars to be used as superior parental lines in combination with reliable molecular markers and high throughput phenotyping techniques, such as the SRI-ToF-MS methodologies presented in this study.

## Data Availability Statement

The raw data supporting the conclusions of this article will be made available by the authors, without undue reservation.

## Author Contributions

BF: conceptualization, investigation, visualization, data analysis, and writing–original draft. IK: investigation, data analysis, and writing–review and editing. MA: texture analysis. FE: investigation and sample preparation. FB: supervision and writing–review and editing. LG: supervision, funding acquisition, and writing–review and editing. All authors contributed to the article and approved the submitted version.

## Funding

This work was financially supported by the AdP of the PAT (Provincia Autonoma di Trento) and partially by the project AppleBerry (L6/99 of the PAT).

## Conflict of Interest

The authors declare that the research was conducted in the absence of any commercial or financial relationships that could be construed as a potential conflict of interest.

## Publisher’s Note

All claims expressed in this article are solely those of the authors and do not necessarily represent those of their affiliated organizations, or those of the publisher, the editors and the reviewers. Any product that may be evaluated in this article, or claim that may be made by its manufacturer, is not guaranteed or endorsed by the publisher.
