# Supplementary material for: Ethylene Production Affects Blueberry Fruit Texture and Storability
Source: Front Plant Sci. 2022 Mar 25;13:813863. doi: 10.3389/fpls.2022.813863 (PMC8990881; doi:10.3389/fpls.2022.813863)
Supplement: Supplementary file 1 [file Data_Sheet_1.docx]

**Supplementary material**

**Supplementary table 1:** List of the *Vaccinium* spp. cultivars employed in this study (HB: Highbush; NHB: Northern highbush; SHB: Southern highbush)

| Cultivar | Species | Type | Harvest date | Pre-harvest | Post-harvest |
| --- | --- | --- | --- | --- | --- |
| Atlantic | *V. corymbosum* | NHB | 26-giu | X | X |
| Berkeley | *V. corymbosum* | NHB | 26-giu | X | X |
| Biloxi | *V. corymbosum, V. virgatum, V. darrowii* | SHB | 26-giu | X | X |
| Bluechip | *V. corymbosum* | NHB | 26-giu | X | X |
| Bluecrop | *V. corymbosum* | NHB | 16-giu |  | X |
| Bluegold | *V. corymbosum* | NHB | 26-giu | X | X |
| Bluetta | *V. corymbosum* | NHB | 16-giu |  | X |
| Brigitta Blue | *V. corymbosum* | NHB | 26-giu | X | X |
| Cabot | *V. corymbosum* | NHB | 16-giu |  | X |
| Chandler | *V. corymbosum* | NHB | 10-lug | X | X |
| Duke | *V. corymbosum* | NHB | 16-giu |  | X |
| Elisabeth | *V. corymbosum* | NHB | 10-lug |  | X |
| Elliott | *V. corymbosum* | NHB | 10-lug |  | X |
| Emerald | *V. corymbosum, V. darrowii, V. elliotti* | SHB | 26-giu | X | X |
| Jersey | *V. corymbosum* | NHB | 26-giu | X | X |
| Jubilee | *V. corymbosum, V. darrowii, V. elliotti* | HB | 26-giu | X | X |
| Legacy | *V. corymbosum, V. darrowii* | HB | 10-lug | X | X |
| Misty | *V. corymbosum, V. darrowii* | SHB | 26-giu | X | X |
| Northblue | *V. corymbosum, V. angustifolium* | NHB | 16-giu |  | X |
| Northland | *V. corymbosum, V. angustifolium* | NHB | 16-giu |  | X |
| Nui | *V. corymbosum* | NHB | 16-giu |  | X |
| Star | *V. corymbosum, V. darrowii, V. virgatum* | SHB | 16-giu |  | X |
| Sunrise | *V. corymbosum, V. angustifolium* | NHB | 16-giu |  | X |
| Toro | *V. corymbosum* | NHB | 16-giu |  | X |


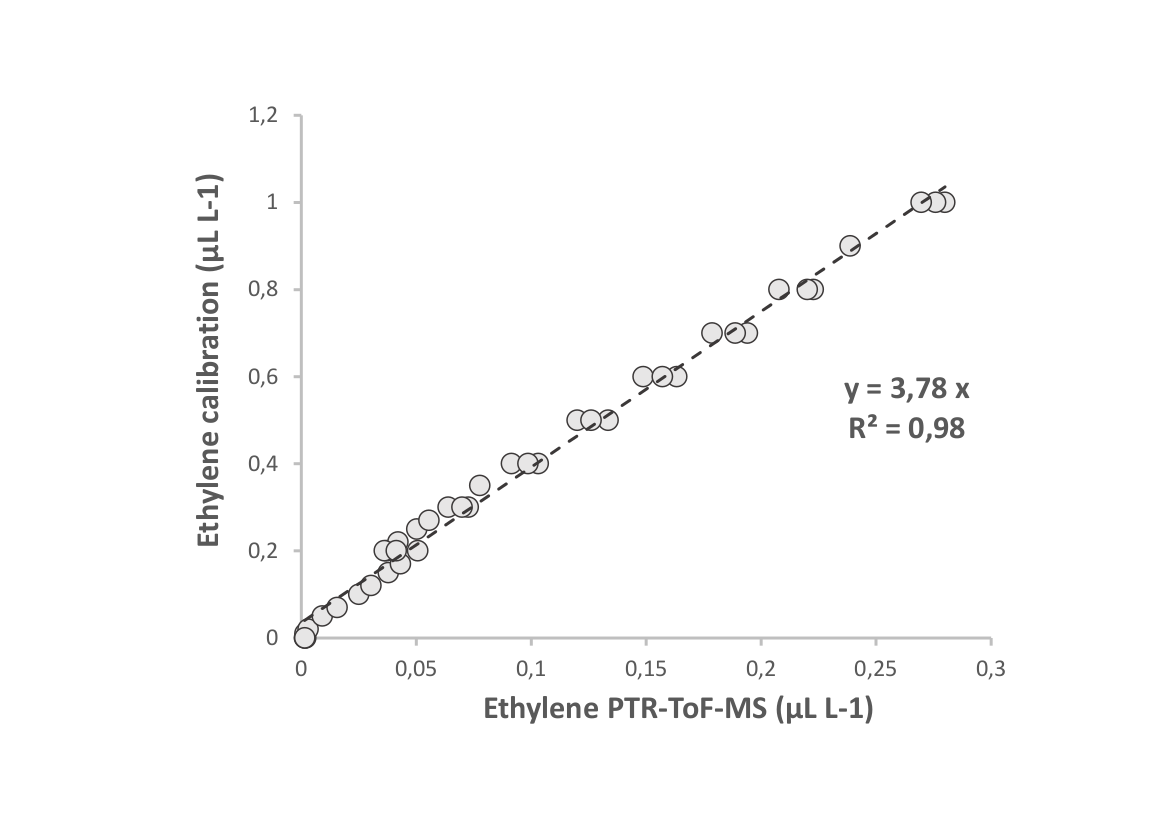


**Supplementary figure 1:** Calibration curve for the ethylene measurement assessed by PTR/SRI-ToF-MS set in O_2_^+^mode.

**
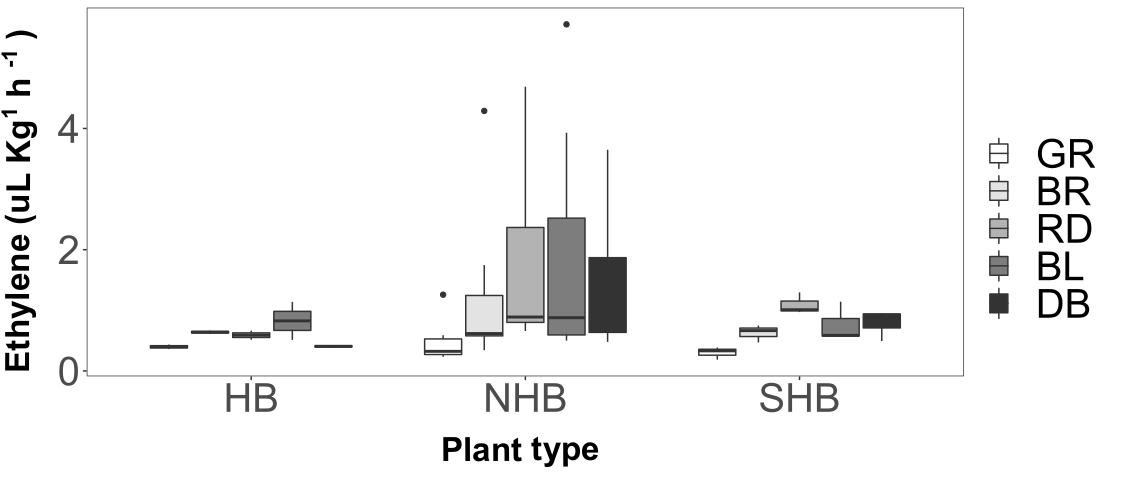
**

**Supplementary figure 2:** box plot of the ethylene production of blueberry during ripening ( green (GR), breaker (BR), red (RD), blue (BL), dark blue (DB) based on the type of plant ( Northern high bush, NHB; Southern high bush, SHB; High bush, HB)

**
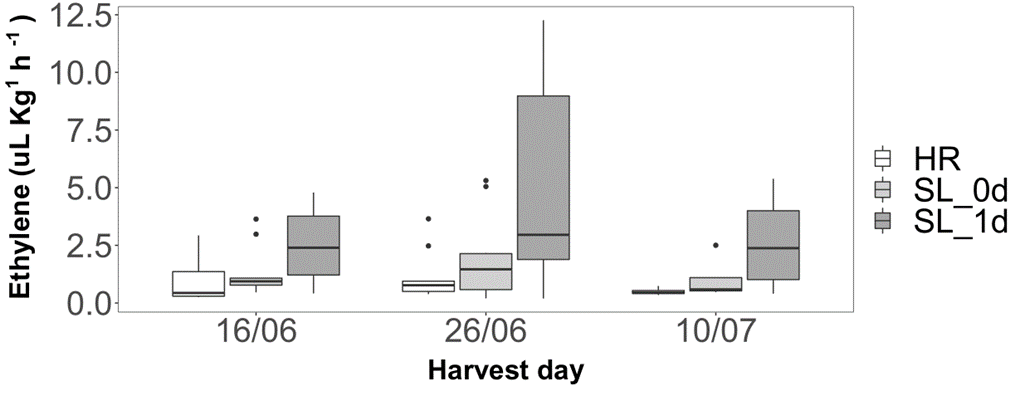
**

**Supplementary figure 3:** box plot of the ethylene production of blueberry during storage based on of the earliness / lateness of the cultivars (harvest times: 16/06; 26/06; 10/07)


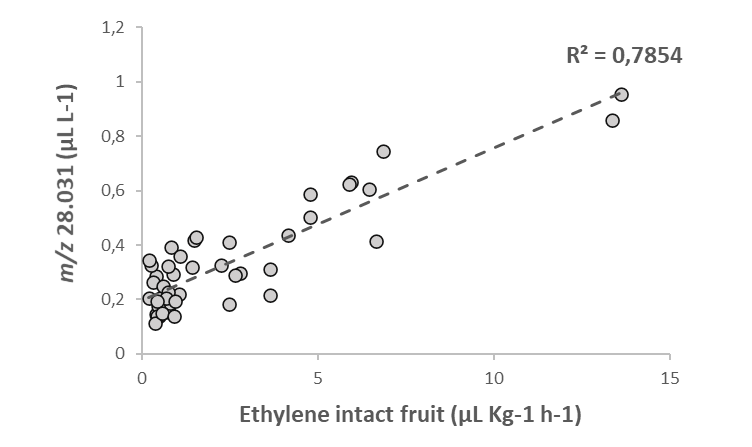


**Supplementary figure 4:** Correlation between the ethylene rate of intact fruit (assessed by PTR/SRI-ToF-MS in O2+mode) and the concentration of m/z 28.031 of frozen powdered fruit (assessed by PTR/SRI-ToF-MS set in H_3_O^+^mode).

**
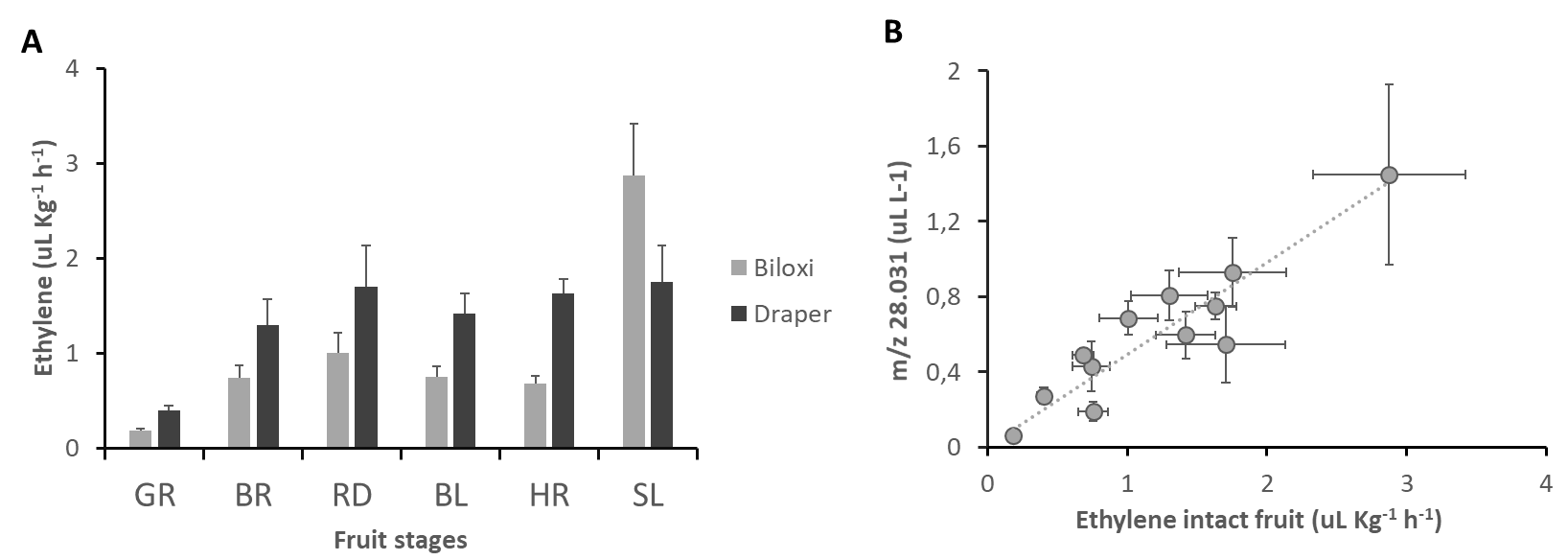
**

**Supplementary figure 5.** Ethylene assessment of the cultivars “Biloxi” and “Draper” during ripening and storage A) Ethylene production rate of fruit of the cultivars “Biloxi” and “Draper” at different ripening storage phases: green (Gr), breaker (Br), red (Rd), blue (Bl), harvest (HR), and after four weeks of storage at 2°C (SL). B) Correlation between the ethylene of intact fruit (assessed by PTR/SRI-ToF-MS in O2+mode) and the concentration of m/z 28.031 of frozen powdered fruit (assessed by PTR/SRI-ToF-MS set in H3O+mode).

**
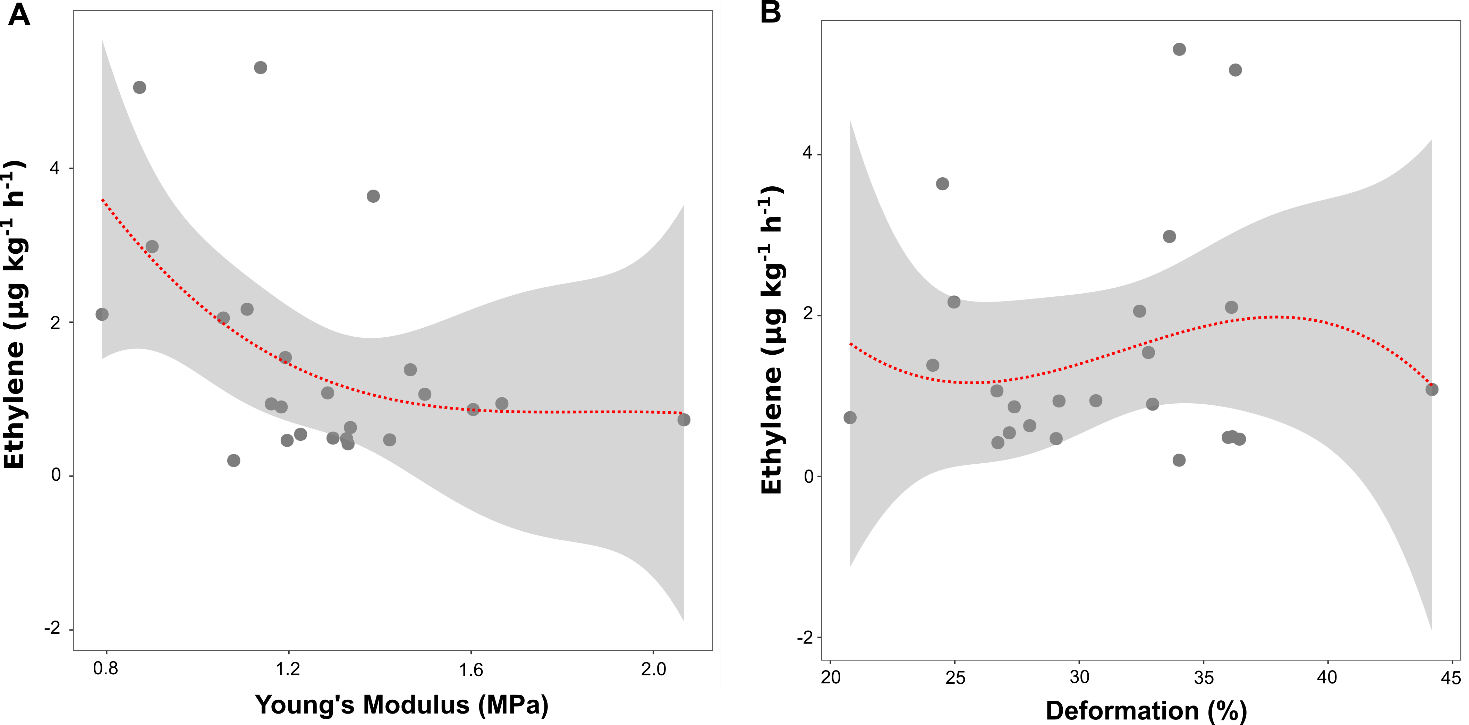
**

**Supplementary figure 6:** Correlation plot and polynomial regression model between the ethylene content and the texture values of Young’s module (A) and deformation at maximum force (B) of 24 blueberry accessions assessed after four weeks of storage. Ethylene measurement was assessed in four replicates on intact berries by using an PTR/SRI-ToF-MS set in O2+mode. Texture analysis was assessed on ten berries by using a texture analyzer.
